# Minimally Invasive Cardiac Surgery: A Health Economic Perspective

**DOI:** 10.31083/RCM53209

**Published:** 2026-10-12

**Authors:** Megan B. Meechem, Kevin Liu, Toshiro WS Sembo, Holly N Smith, Daniel D Holloway, Muhammad R Ahsan, Ganesh Shanmugam, Bob Kiaii, William DT Kent, Corey Adams, Ali Fatehi Hassanabad

**Affiliations:** ^1^Libin Cardiovascular Institute, University of Calgary, Calgary, AB T2N 1N4, Canada; ^2^Department of Physiology and Pharmacology, Cumming School of Medicine, University of Calgary, Calgary, AB T2N 1N4, Canada; ^3^Division of Cardiac Surgery, Department of Cardiac Sciences, Libin Cardiovascular Institute, Cumming School of Medicine, University of Calgary, Calgary, AB T2N 1N4, Canada; ^4^Section of Cardiac Surgery, Department of Surgery, Columbia University Medical Center, New York Presbyterian University, New York, NY 10032, USA

**Keywords:** health economics, cardiac surgery, minimally invasive cardiac surgery

## Abstract

This study aimed to review the contemporary literature on the health economics of minimally invasive cardiac surgery (MICS). MICS is being increasingly adopted worldwide. While the clinical and patient-reported outcomes of MICS have been established, uncertainty remains regarding the health economic aspect of MICS. This review aims to address that knowledge gap. This is a comprehensive narrative review of articles evaluating the health economics of MICS across various jurisdictions and healthcare systems. Electronic databases, including PubMed, Embase, and Scopus, were searched from inception through November 2025. Search terms included combinations of MICS, health economics, minimally invasive direct coronary artery bypass, mini-aortic valve replacement, mini-thoracotomy, hemi-sternotomy, aortic valve replacement, right anterior mini thoracotomy, robotic cardiac surgery, robot-assisted cardiac surgery, video-assisted cardiac surgery, and port-access cardiac surgery. Across procedure types, studies consistently demonstrated that high-volume, well-standardized MICS programs can achieve cost neutrality or cost savings, relative to conventional surgery, with reported hospital cost reductions ranging from approximately 9% for mini-aortic valve replacement to CAD 16,700 for minimally invasive mitral valve surgery. These savings are largely attributable to improved postoperative efficiency and reduced resource utilization. In contrast, centers with low surgical volumes or fragmented workflows often incur significantly higher costs per case and risk underutilizing expensive technologies, such as robotic systems. When implemented in well-resourced, experienced centers, MICS appears to be a promising, value-driven strategy for contemporary cardiac care. Health systems should consider centralizing MICS services to optimize capital investment and realize sustainable economic value, rather than distributing these resources broadly across lower-volume centers.

## 1. Introduction

Health economics offers a framework for assessing resource allocation, innovations, and the impact of interventions on costs, outcomes, and value for patients and society [1]. Economic assessments are important in healthcare because it guides how limited resources can be used most efficiently to improve patient outcomes, support equitable access, and ensure that health systems deliver the greatest value for the populations they serve [2,3]. In cardiac surgery, one of the most resource-intensive areas of medicine, health-economic evaluations are essential to understanding how interventions affect both clinical outcomes and the sustainability of healthcare delivery. Globally, economic analyses increasingly shape decisions surrounding new technologies and surgical approaches. However, within the Canadian healthcare system, there remains a relative scarcity of robust health-economic studies in Cardiac Surgery. Given Canada’s publicly funded structure, finite operating room capacity, and emphasis on equitable care distribution, generating high-quality economic data is critical to supporting evidence-based policy, optimizing resource allocation, and guiding the adoption of emerging surgical strategies.

Minimally invasive cardiac surgery (MICS) has emerged as a transformative approach in the surgical management of cardiovascular disease, offering comparable or superior clinical outcomes to conventional median sternotomy while potentially reducing perioperative morbidity, hospital length of stay, and recovery time [4,5,6]. Over the past two decades, there has been a significant increase in the number of MICS performed globally. Technological advancements, improved imaging modalities, and refined surgical techniques have facilitated the expansion of MICS for procedures such as valve repair or replacement, coronary revascularization, and atrial septal defect closure [4,7,8,9]. Clinically, MICS has been associated with reduced postoperative pain, shorter intensive care unit (ICU) and hospital stays, improved cosmetic outcomes and patients experiencing a quicker return to typical daily activities [10,11,12,13]. These advantages have positioned MICS as an attractive surgical alternative for both patients and healthcare systems. However, its widespread adoption has been tempered by concerns regarding cost-effectiveness, procedural complexity, and resource utilization.

Despite the clinical advantages, the economic implications of MICS remain a subject of ongoing debate. From a health economics perspective, MICS presents a nuanced balance between higher upfront expenditures, driven by specialized instrumentation, longer operative times, and extensive surgical training and potential downstream savings resulting from shorter ICU stays, faster rehabilitation, and reduced readmission rates [14,15]. Economic evaluations are further complicated by heterogeneity in healthcare delivery models, reimbursement frameworks, and institutional experience, resulting in substantial variation in cost-effectiveness across healthcare centers and regions. Therefore, understanding the true economic value of MICS requires a comprehensive assessment that integrates short- and long-term costs, quality-adjusted life years (QALYs), and system-level outcomes. Yet, the existing literature is scarce, with most studies limited by short follow-up durations, inconsistent cost definitions, and variable inclusion of indirect societal costs such as lost productivity.

This comprehensive narrative review aims to synthesize current evidence on the health economics of MICS, evaluating its cost-effectiveness relative to conventional cardiac surgery and identifying the methodological and systemic factors that influence economic outcomes. By critically examining direct and indirect costs, resource allocation, and long-term patient-centered outcomes, this work seeks to inform future policy, clinical decision-making, and research directions in the pursuit of economically sustainable cardiac care.

## 2. Methods

A comprehensive literature search was conducted to identify studies evaluating the health economics of MICS compared with conventional cardiac surgery. Electronic databases including PubMed, Embase, and Scopus were searched from their inception until November 2025. Search terms included combinations of Medical Subject Headings (MeSH) terms and keyword variations for MICS, health economics, costs, minimally invasive direct coronary artery bypass (MIDCAB), minimally invasive aortic valve replacement (mini-AVR), mini thoracotomy, hemi-sternotomy, aortic valve replacement (AVR), right anterior mini thoracotomy (RAMT), robotic cardiac surgery, robot-assisted cardiac surgery, video assisted cardiac surgery and port-access cardiac surgery.

The aim of this review was to provide a narrative, expert-driven overview of the current literature on MICS. Relevant publications were identified through targeted searches of the aforementioned databases. The scope of this review was to provide a comprehensive overview of key existing clinical and economic evidence. Findings were synthesized, integrating published evidence with interpretation to highlight current knowledge, emerging trends, and areas of uncertainty or ongoing discussion.

## 3. Health Economics in Surgical Innovation and Cardiac Surgery

### 3.1 Conceptual Framework of Health Economics in Surgical Innovation

Across surgical subspecialties, health economics provides a framework for evaluating whether healthcare innovations will improve patient outcomes while making efficient use of finite healthcare resources. In disciplines such as general, thoracic, gynecologic, orthopedic, and vascular surgery, health economic analyses have increasingly guided whether healthcare systems should adopt innovative approaches when they deliver meaningful system-level benefits, even when the upfront procedural costs are higher [16,17,18]. Thus, health economics in this context has shifted from solely focusing on technical success towards a broader basis of consideration including clinical effectiveness, patient-centred outcomes, and economic sustainability.

Health economics’ core consists of recognizing that healthcare resources are limited which necessitates decisions that maximize health gains while minimizing unnecessary expenditures. This can be explored through several economic concepts. Direct costs address expenditures that are directly associated to patient care, including operative equipment, disposable surgical instruments, healthcare personnel, intensive care/hospital stay and resources, medications, and postoperative management. Direct costs can be further divided into fixed costs and variable costs. Fixed costs include finances such as capital investment in robotic technology or minimally invasive surgical infrastructure. Variable costs are those that can change, either increase or decrease, with each additional procedure.

Marginal cost is the additional cost that is incurred by performing one extra procedure. It represents the incremental resources required for each additional case performed and is calculated by the change in total cost divided by the change in procedural volume. In the context of MICS, marginal costs are primarily associated with procedure-specific consumables, operating room time, perfusion resources, postoperative care, and hospital lengths of stay. As surgical programs and practices mature and become more efficient, marginal costs often decline, making this concept notably relevant when evaluating economic implications when implementing MICS at healthcare institutions.

Another crucial principle is opportunity cost, which is associated with the cost of healthcare resources spent when one intervention is chosen instead of the next best alternative. Since hospital resources are finite, investing in one surgical strategy will inevitably limit the availability of these resources for other patients/services. As such, adopting novel surgical strategies like MICS should be assessed based not only on their absolute costs, but also take into consideration the potential for these strategies to generate greater health benefits than competing strategies using the same resources. Economic evaluations also require standardized measures that integrate postoperative survival length and postoperative quality of life [19]. The quality-adjusted life year (QALY) is the most universally utilized metric to reflect this, which combines life expectancy with health-related quality of life into a single measurement of health benefit. QALYs are calculated by multiplying the duration of survival by a health utility value ranging from 0 (death) to 1 (perfect health). By doing so, this allows for comparison of interventions across different diseases and surgical procedures/specialties.

The learning curve effect is an economical concept that is also relevant to surgical innovation because of the nature of increasing procedural experience leading to progressive improvements in operative efficiency and patient outcomes. Economic evaluations in surgical innovation often quantifies the learning curve through measuring cumulative metrics such as procedural volume, operative duration, segmented regression, or experience-adjusted statistical models. These quantification methods allow for identification of the point at which procedural performance stabilizes. Thus, as surgical teams gain proficiency, this will allow for estimations of at what point one can expect to see reductions in operative time, complication rates, ICU/hospital lengths of stay, and overall costs.

The economic concepts described in this section provide a basis for what formal health economic evaluation would consist of. Together, these approaches provide complementary perspectives to determine if MICS has the potential to act as an efficiency use of healthcare resources.

### 3.2 Health Economics in Cardiac Surgery

Health economic principles are particularly relevant in Cardiac Surgery, associated with some of the most resource-intensive procedures across all fields of surgical practice. When comparing any two specific specialties, however, there is still a considerable amount of variability in the robustness of economic evidence. Differences in patient characteristics, procedural complexity, surgical volume, and standardization of care pathways can influence the feasibility and rigor of economic analysis [20]. As a result, while some areas of surgery have developed mature cost-effectiveness data, others, such as Cardiac Surgery and MICS continue to face notable evidence gaps (Fig. 1). These discrepancies underscore the need for ongoing, specialty-specific health economic research to ensure that new technologies and techniques are adopted responsibly, cost-effectively, and with maximal impact on both patient outcomes and healthcare system performance. In particular, with Cardiac Surgery representing a field of medicine where increasingly complex interventions meet constrained healthcare resources, even modest improvements in clinical efficiency or reductions in postoperative morbidity can translate into substantial system-level economic gains [21,22,23]. Thus, economic evaluations in Cardiac Surgery extend beyond sole comparisons of procedural costs. Comprehensive assessments span across three major domains: direct procedural costs, postoperative resource utilization, and long-term cost and quality of life effectiveness as assessed through metrics like QALYs.

**Fig. 1.**
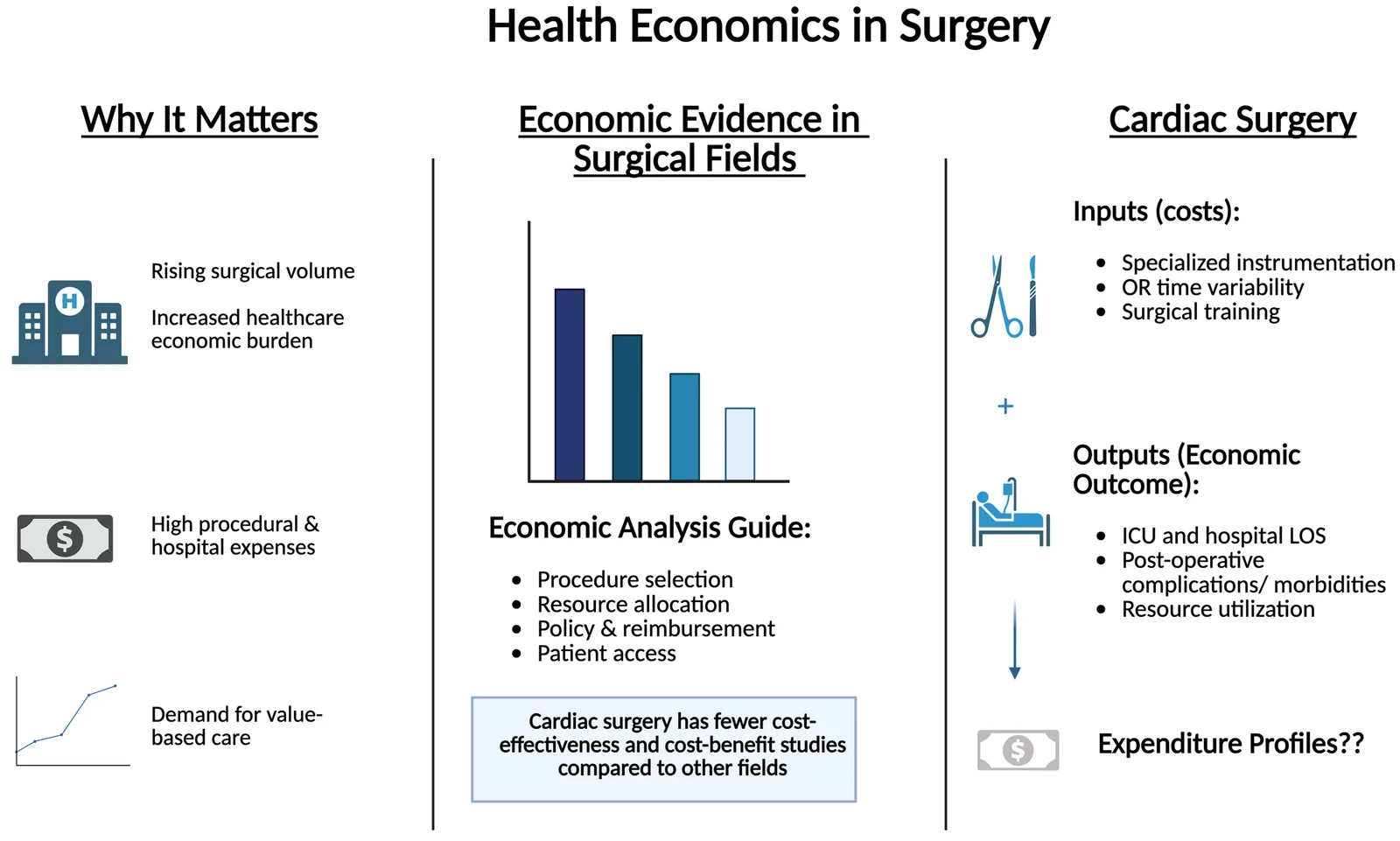
**Illustration emphasizing the need for health-economic assessments across surgical specialties, outlining their broad influence on healthcare delivery, and highlighting the scarcity of cost-analysis studies in cardiac surgery despite its substantial contribution to overall healthcare expenditures**. Schematic generated using BioRender.

## 4. Health Economics in Minimally Invasive Cardiac Surgeries (MICS)

Health economics is particularly relevant to MICS, where the balance between higher procedural costs and potential downstream savings must be clearly defined. While MICS often requires specialized equipment, longer operative times, and additional training, these upfront investments may be offset by shorter hospital stays, decreased postoperative complications, reduced ICU utilization, and faster recovery. Economic evaluations, primarily from international centers with established MICS programs, suggest that despite higher initial expenditures, MICS may deliver comparable or superior value relative to conventional sternotomy [24,25]. However, the extent to which these findings translate to different healthcare systems, including Canada’s publicly funded model, remains uncertain, underscoring the need for context and regionally specific health-economic analyses. The following sections examine how these health-economic principles manifest within specific minimally invasive cardiac surgery domains, including minimally invasive and robotic coronary artery bypass procedures, as well as minimally invasive aortic and mitral valve surgeries (Table 1, Ref. [15,26,27,28,29,30,31,32,33,34,35,36,37,38,39]). In subsequent subsections of this review, the term “cost” refers to distinct economic constructs depending on the source study. These include intraoperative procedural costs, index hospitalization costs, total episode of care costs incorporating post-discharge events such as readmissions and rehabilitation, and societal costs such as productivity loss of the patient. Since published studies have variation in the cost components included, direct comparisons between studies mentioned in this review should be interpreted with caution.

**Table 1.** **Health economic findings across minimally invasive cardiac surgery (MICS) procedures**.

MICS procedure type	Study designs	Perspective	Cost comparison (MICS vs. conventional)	Drivers of higher upfront cost	Downstream savings / economic benefits	References
MIDCAB	Retrospective cohort studies	Hospital/provider	Typically, higher intraoperative costs; becomes cost-neutral or cost-saving in high-volume programs.	Specialized instruments Large learning curve and training. Longer operating time during early adoption.	Reduced length of stay in hospital, fewer complication and faster recovery times	[26,27,28]
RCAB	Observational, perspective review	Payer/health system	High upfront costs: centralized procurement and high case volume reduces per-case cost substantially.	Robotic capital acquisition (~USD 1–2.5M) and annual maintenance and disposable supplies (~100–170k/year USD).	Lower per-case costs achieved through high volumes and centralized healthcare systems. Economic savings only achieved when robotic systems are heavily utilized (>100–150 cases/year).	[29,30]
Mini-AVR (Hemi-sternotomy AVR)	RCT, retrospective studies, cohort observational studies	Hospital, societal	Some observational studies have reported reductions in postoperative costs; however, overall cost-effectiveness or economic superiority of mini-AVR has not been consistently demonstrated, particularly based on higher-quality evidence.	Slightly longer cross-clamp and bypass times. Longer discharge readiness time.	Reduced ICU stay and fewer complications. No reduction in transfusion or length of stay. Minimal clinical differences. ~ 9.3% reduction in postoperative costs.	[31,32,33,34,35]
Mini-AVR (Right Anterior Mini Thoracotomy – RAMT)	Retrospective cohort studies	Hospital/provider	Lower total hospital costs (38,769 USD vs 42,656 USD)	Specialized access, surgical training and instrumentation.	Shorter ICU stay, less patients requiring transfusion and shorter hospital length of stay.	[36,37,38]
MIMVS	Observational, review	Hospital/provider	MIMVS procedure with rapid recovery protocol is economically beneficial. Robotic MIMVS becomes cost-saving with optimized workflows Consistent direction toward system-level cost savings in high-performing centres	Specialized equipment; longer operating room times in early adoption of technique. High costs associated with robotic capital and disposables.	Major savings in ICU/ward stay. Lower postoperative cost for MIMVS: 23,366 CAD vs 40,111 CAD.	[15,39]

Mini-AVR, minimally invasive aortic valve replacement; MIDCAB, minimally invasive direct coronary artery bypass; RCAB, robotic-assisted coronary artery bypass; MIMVS, Minimally Invasive Mitral Valve Surgery; ICU, intensive care unit.

### 4.1 Coronary Surgery: Minimally Invasive (MIDCAB) and Robotic Coronary Artery Bypass (RCAB)

MIDCAB and RCAB surgeries have been developed as alternatives to conventional sternotomy for selected patients requiring coronary revascularization. These approaches aim to reduce surgical trauma while maintaining the efficacy of conventional coronary artery bypass grafting (CABG), particularly in patients with isolated left anterior descending (LAD) coronary artery disease or limited multivessel disease [40,41].

Clinically, MIDCAB and RCAB procedures provide comparable or superior perioperative outcomes relative to conventional CABG while potentially improving patient satisfaction through reduced postoperative pain, shorter recovery times, and smaller surgical scars [42,43]. Despite the well-known clinical benefits, from an economic perspective, these minimally invasive procedures may be more cost-effective in high-volume medical centers where operative efficiency and surgeon experience reduce the incremental costs of robotic equipment and minimally invasive techniques. Due to specialized equipment, prolonged operative durations during implementation and extensive technical training, the initial operative costs are typically higher for MIDCAB and RCAB surgeries [44]. The significant upfront capital investment of robotic cardiac equipment is a primary contributor to the elevated costs associated with RCAB procedures. Additionally, the robotic machinery requires regular service contracts, maintenance and surgery-specific disposable supplies [45].

To justify the substantial equipment-affiliated costs of these procedures, hospitals often utilize either straight-line, annuitized, or activity-based amortization models to disperse the expense over the duration of the system’s expected lifespan and predicted case-volume [46]. Previous studies have shown that the structure of the healthcare system can impact the economic expenditures. Global healthcare systems, such as the NHS, that use centralized procurement models can reduce the acquisition and maintenance costs by purchasing specialized equipment in large quantities, standardizing physician training and consolidate cases at specialized, high-volume centers, which lowers the costs per procedure using robotic systems [29,47]. A nationwide UK NHS study conducted by Lam et al. [29] found that between 2013 and 2019 the average surgical robotic machinery acquisition cost was £1,350,000, with an additional expense of £140,000 for the annual disposable supplies and maintenance fees. Despite the elevated upfront costs this study found that centralized procurement and higher case-volumes for RCAB procedures significantly decreased the per-procedure costs [29].

In contrast, healthcare systems that use hospital-specific purchasing models, such as the United States, have the benefit of regional control and operational flexibility but these advantages result in an economic trade-off of inflated individual case costs and an increased risk of under-utilization of resources [48]. For instance, Barbash and Glied’s national economic assessment of robot-assisted surgery demonstrated that many US hospitals purchased robotic platforms independently to maintain local control and competitive positioning; however, this autonomy often resulted in substantial under-utilization, with numerous medical institutions performing less than 100 robotic cases annually [30]. At these volumes, the fixed capital costs, ranging from USD 1–2.5 million for acquisition and USD 100,000– $ 170,000 per year for maintenance, were inefficiently amortized, driving per-procedure costs several thousand dollars higher than at high-volume institutions. Secondary to the fixed and variable costs associated with MIDCAB and RCAB surgeries, achieving a health system with relative economic neutrality is highly dependent on numerous factors, including procedural volume, reduced surgical complications and faster postoperative recovery times [28,29,30,49].

It is important to note that studies like Barbash and Glied’s national economic assessment was conducted in the early 2000s. While very robust and well accredited, the economy has nonetheless drastically changed over the past decade. More recent evidence suggests that economic evaluation of robotic cardiac surgery has evolved beyond the cost analyses that were performed at the outset of the adoption of robotic systems. A study by Patrick et al. from 2021 [44] demonstrated that increasing surgeon experience at healthcare institutions with RCAB was associated with improved operative efficiency, underscoring the learning curve effect when interpreting procedural costs [30]. Stringfield et al. in 2022 [45] reported that across a 10-year period, robotic surgery programs of higher maturity and more established standardized workflows improved resource utilization ultimately reducing the impact per-case of fixed capital expenditures [31]. Lam et al. in 2021 [29] further demonstrated that centralizing or regionalizing the implementation of robotic surgery within the United Kingdom National Health Service improved utilization and reduced the maintenance costs per-procedure [33]. Interestingly and in comparison, Shen et al. [50] reported that in the United States, adoption of robotic surgery in healthcare institutions is often heavily influenced by institutional and competitive factors rather than solely from economic efficiency alone.

Overall, when full episode-of-care costs are considered, minimally invasive or robot-assisted approaches can be economically viable strategies, particularly in high-volume, well-established programs, or centralized healthcare systems. Lower incremental costs per case due to economic healthcare profiles and scale, including volume and cross-specialty utilization, enhance procedural value. MIDCAB and RCAB represent economically viable strategies for selected patients when case and healthcare system-specific costs are considered.

### 4.2 Minimally Invasive Aortic Valve Surgery

Although minimally invasive aortic valve replacement (mini-AVR) may incur higher intraoperative expenditures, such as longer operative times, increased perfusion duration, or specialized equipment, multiple observational studies have reported that these upfront costs can be offset by postoperative efficiencies such as decreased transfusion requirements, shorter ICU utilization, and reduced overall length of stay [37,51]. However, randomized trials and recent systematic reviews indicate that the magnitude and consistency of these economic benefits vary considerably by institution, technique, and regional health-system structure. The following subsections synthesize the economic and clinical evidence for the two principal mini-AVR techniques: hemi-sternotomy and RAMT.

#### 4.2.1 Hemi-Sternotomy for Aortic Valve Replacement

Hemi-sternotomy (upper partial sternotomy) remains one of the most widely adopted minimally invasive access routes for surgical AVR. It offers excellent operative exposure with fewer specialized instrumentation requirements compared with thoracotomy approaches, contributing to its use across diverse surgical programs. Several observational cost analyses have suggested meaningful economic advantages for hemi-sternotomy. For example, a cost-benefit evaluation by Sanad and Beshir comparing T-shaped hemi-sternotomy with full sternotomy across two Egyptian centers reported a postoperative cost reduction of approximately 9.3%, primarily driven by shorter ICU stays and fewer postoperative complications [35]. Interestingly, contemporary randomized evidence has not consistently demonstrated overall cost effectiveness of MICS compared to conventional procedures. Some examples of this would be a randomized controlled trial by Hancock et al. [31] showing that while perioperative clinical outcomes were broadly comparable between groups, mini-sternotomy was associated with longer cardiopulmonary bypass and aortic cross-clamp times and did not reduce transfusion requirements. When these data were incorporated into an NHS-based cost-effectiveness model, mini-sternotomy did not produce lower overall treatment costs or improved quality-adjusted life-years. Indeed, conventional sternotomy demonstrated a higher probability of being cost-effective at standard UK willingness-to-pay thresholds [31]. Similar findings were observed in the *Mini-Stern* randomized trial, where long-term follow-up of randomized participants also demonstrated no survival or reoperation advantage for the minimally invasive approach [32]. Additionally, the 2023 Cochrane review concluded that while hemi-sternotomy may modestly reduce blood loss, high-quality evidence does not demonstrate consistent improvement in ICU stay, hospital stay, complications, or cost-effectiveness [52].

#### 4.2.2 Failure of Hemi-Sternotomy to Achieve Cost-Effectiveness

Hemi-sternotomy is typically seen as the least technically complex minimally invasive approach for surgical aortic valve replacement. Since the clinical advantages associated with hemi-sternotomy appear to be relatively modest, this may render randomized trials to demonstrate inconsistency in showing meaningful superiority over conventional procedures. As a result, the QALYs generated by hemi-sternotomy become minimal and can limit the ability for hemi-sternotomy to justify higher procedural expenditures.

As mentioned in the previous section, multiple randomized studies reported longer cardiopulmonary bypass and aortic cross-clamp times associated with mini-sternotomy. This thereby increases other costs such as operating room expenditures, perfusion costs, and anesthetic/surgical personnel costs. In other words, the marginal cost of each procedure increases with hemi-sternotomy. However, it is important to note that corresponding postoperative savings offset a significant portion of the increase in marginal cost, but evidently not enough to consistently demonstrate superiority of MICS over conventional procedures. Another health economic concept that should be considered is the opportunity cost of hemi-sternotomy. Longer operating room occupancy for procedures that seemingly provide little additional benefit may reduce operating room throughput and constrain access for other surgical cases. As such, resources devoted to hemi-sternotomy may generate less overall health gain than alternative investments, especially when conventional sternotomy potentially achieves comparable clinical outcomes at lower overall cost.

This section demonstrates that beneficial findings reported in current literature about hemi-sternotomy may be too small to compensate for the increased procedural costs associated with longer operative times and greater resource utilization. This contrasts with some other sections in this review, which in some cases demonstrate larger reductions in postoperative morbidity, ICU/hospital stay, and recovery time may produce sufficient downstream savings to offset the uneconomical nature of MICS.

#### 4.2.3 Right Anterior Mini Thoracotomy Aortic Valve Replacement

RAMT has emerged as an alternative minimally invasive technique for AVR, providing direct access to the aortic valve through a limited right anterior thoracic incision while avoiding full sternotomy [53,54]. Despite its increasing use, economic evaluations of RAMT remain more limited.

A large multi-institutional analysis demonstrated that RAMT was associated with significantly lower mean total hospital costs than full sternotomy AVR (38,769 vs. 42,656 USD; *p* < 0.01), with cost savings driven primarily by reduced transfusion needs, shorter ICU stays, and abbreviated overall hospitalization [36]. Furthermore, comparative cost modeling between mini-sternotomy AVR and mini-thoracotomy AVR indicated that while clinical outcomes are broadly similar between the two minimally invasive techniques, procedure-related costs vary substantially. For an annual volume of 50 cases, the modeled incremental average cost differences compared to full sternotomy AVR procedures were 290 USD for mini-sternotomy and 4254 USD for mini-thoracotomy [38]. These findings highlight that economic performance of minimally invasive approaches is highly sensitive to institutional factors such as annual case volume, perioperative workflow optimization, and surgeon experience.

Taken together, the economic performance of minimally invasive AVR is highly context dependent. Observational studies, particularly from high-volume minimally invasive programs, frequently report reductions in ICU use, postoperative complications, and overall length of stay for hemi-sternotomy and RAMT approaches. However, the highest-quality evidence, including two UK randomized controlled trials and a Cochrane review, has not demonstrated consistent economic advantages for hemi-sternotomy over full sternotomy, and cost-effectiveness varies substantially across healthcare systems. RAMT may offer cost savings in selected centers, though existing data remain limited and predominantly observational. As such, economic conclusions must be interpreted within each healthcare institution’s operative efficiency, case volume, resource expenditures, and surgeon experience.

### 4.3 Minimally Invasive Mitral Valve Surgery (MIMVS)

MIMVS has emerged as a viable alternative to conventional median sternotomy for the correction of degenerative and functional mitral valve disease. By utilizing smaller lateral thoracotomy incisions and video- or robotic-assisted instrumentation, MIMVS aims to reduce operative trauma while preserving procedural efficacy. Similarly to other MICS procedures, MIMVS has been associated with reduced perioperative complications, less postoperative pain, shorter ICU and hospital durations, and faster return to functional status [4,55]. These patient-centered advantages have contributed to increasing adoption; however, from a health economics perspective, MIMVS presents a complex and multifaceted cost profile.

Similar to MIDCAB and RCAB procedures, the direct intraoperative costs tend to exceed those of conventional open mitral valve repairs, however, these higher upfront costs may be offset by downstream savings such as reduced postoperative complications, accelerated recovery trajectories, and decreased length of stay. Regional economic evaluations support this nuanced cost structure, showing mixed but generally favorable findings regarding MIMVS cost-effectiveness. Several cost analysis reports indicated that compared to conventional approaches the procedural expenditures for MIMVS are higher, however total hospital costs can become balanced or even marginally reduced due to decreased ICU and ward utilization [56,57]. A retrospective study conducted at the Foothills Medical Centre in Alberta, Canada, found that the mean postoperative cost per MIMVS patient was 23,366 CAD, whereas the average postoperative cost per open mitral valve procedure, undergoing a median sternotomy, was approximately 40,111 CAD [15]. Furthermore, another Canadian cost analysis conducted in Quebec reported that the robotic MIMVS approach was associated with an additional 3444 CAD per procedure when amortized equipment costs were included [58]. However, the same study found that integrating robotic MIMVS with optimized operating room efficiency and standardized postoperative care resulted in an overall cost reduction compared to an open sternotomy (30,606 CAD vs. 31,310 CAD) [58].

Collectively, these clinical and economic findings suggest that MIMVS demonstrates a consistent trend toward reducing perioperative resource utilization without compromising surgical outcomes. When the full episode of care is considered, MIMVS may be economically beneficial. Moreover, broader adoption and increased surgeon experience may further amplify these economic benefits by reducing operative times and improving workflow efficiency. Therefore, as health systems work to minimize expenditures while maintaining high-quality cardiac care, understanding the balance between upfront resource utilization and downstream savings is essential for determining if MIMVS delivers optimal value.

## 5. Perspective

Across the literature, MICS is consistently associated with increased upfront and intraoperative costs relative to conventional median sternotomy approaches. A substantial portion of this cost differential stems from the reliance on advanced technologies, particularly robotic and specialized imaging platforms, that remain in an intermediate stage of development: sophisticated enough to warrant clinical integration yet not economically efficient global adoption [50,59,60]. This developmental phase contributes to substantial capital, maintenance, and servicing costs, especially for systems such as the Da Vinci robotic platform, which impose approximately 3000-4000 USD in amortized cost per case, even in high volume centers, which contributes to the markedly higher financial burden on institutions compared with traditional operative approaches [46,61,62].

Beyond equipment, MICS also necessitates investment in the institutional program development, including dedicated perfusion circuits, long-shafted instruments, port-access cannulation equipment and advanced visualization tools, all of which elevate baseline expenditures [63,64,65]. The technical complexity of operating through limited incisions prolongs cardiopulmonary bypass and cross-clamp times, representing an additional driver of intraoperative cost inflation [66,67,68,69]. The substantial technical training and prolonged learning curve required for surgeons to perform MICS safely and effectively represent an additional source of upfront cost, as institutions must invest considerable time and resources to develop and maintain procedural proficiency. Taken together, these factors explain the robust agreement across studies that intraoperative expenditures are significantly elevated in MICS cases.

Despite higher upfront costs, a consistent finding across economic evaluations is that postoperative savings for most MICS procedures frequently offset, and in some cases surpass, the initial financial burden. Reduced ICU and hospital lengths of stay remain the largest contributors to downstream savings [39,70,71]. Because MICS avoids a full median sternotomy, patients tend to recover faster and require fewer days of intensive staffing resources from nurses, respiratory therapists, physicians, physiotherapists, and allied health professionals. Lower postoperative morbidity further contributes to cost containment. MICS is associated with reduced rates of arrhythmias, renal dysfunction, and infections, resulting in fewer interventions, diminished resource use, and lower complication-related expenditures [72,73,74]. Additionally, decreased blood loss and reduced transfusion requirements further narrow the economic gap between minimally invasive and conventional procedures [75]. In a 1304-patient multi-institutional mini-mitral valve repair cohort, minimally invasive cases demonstrated shorter ICU stays (24 vs 29 hours), shorter hospitalization (4 vs 5 days), and fewer transfusions, resulting in total hospital costs similar to or lower than sternotomy despite longer operative times [76]. Similar trends appear in cost-effectiveness studies of mini thoracotomy mitral valve surgery, where lower postoperative morbidity, fewer infections, fewer laboratory and imaging expenditures, and shorter hospital stays collectively generated cost reductions of approximately 4500–9000 USD per patient [39,77].

Accelerated recovery protocols further amplify this effect; one European program implementing enhanced recovery after surgery (ERAS) in MICS valve surgery demonstrated a reduction in length of stay by 1.6 days and cost savings of nearly €2000 per case [71]. Additionally, from a broader societal perspective, MICS appears to facilitate faster return to independent mobility, daily activities, and employment. Improved functional recovery translates into productivity gains that extend beyond hospital budgets, supporting the argument for considering societal-level cost savings in future economic analyses [4]. Collectively, these findings illustrate a consistent trend: although MICS demands higher initial investment and often incurs longer operative times, the substantial reductions in postoperative resource use, including ICU hours, hospital length of stay, complication rates, and transfusion requirements remain the primary drivers of cost savings. These patterns emphasize that the economic value of MICS cannot be assessed solely through operative costs; rather, it emerges through the cumulative efficiencies achieved across the entire perioperative and recovery continuum.

A recurring theme in the literature is the critical influence of institutional experience and surgical case volume. High-volume institutions with standardized MICS protocols consistently demonstrate shorter operative durations, less perioperative complications, improved postoperative recovery metrics, and more favorable cost profiles [7,29,30,34,47,78]. These findings underscore that MICS cost-effectiveness is not solely determined by technology but is heavily dependent on program maturity, team proficiency, perioperative workflow optimization, and investment in standardized care pathways. Conversely, centers with low annual MICS volumes or fragmented workflows face a heightened risk of inflated individual-case costs and underutilization of expensive equipment, undermining economic justification [79,80]. This variability highlights the importance of regional planning, coordinated resource allocation, and targeted MICS implementation in settings capable of sustaining adequate procedural volumes. Moreover, the economic justification for MICS varies markedly by jurisdiction, driven by differences in healthcare funding models, hospital budgeting structures, labour costs, and reimbursement frameworks. In resource-limited settings, where capital investment is constrained, careful assessment of procedural volume projections, surgical training opportunities, and long-term sustainability is essential to avoid costly underutilization.

Although these themes were not directly evaluated in this literature review, several additional advantages of MICS merit acknowledgement, as they suggest that its value extends beyond strictly measurable cost savings. Patients often report higher satisfaction following MICS due to improved cosmetic outcomes, reduced postoperative discomfort, and a faster return to daily activities or employment [81,82,83]. Shorter hospital stays also create opportunities for increased institutional throughput, allowing health systems to treat more patients within existing resource constraints. Furthermore, MICS has been associated in some series with lower rates of postoperative complications, which may further reduce downstream resource use (Fig. 2). While these broader clinical and societal benefits are promising, formal, context-specific economic evaluations are required to determine the extent to which they translate into quantifiable financial savings within different global and regional healthcare systems.

**Fig. 2.**
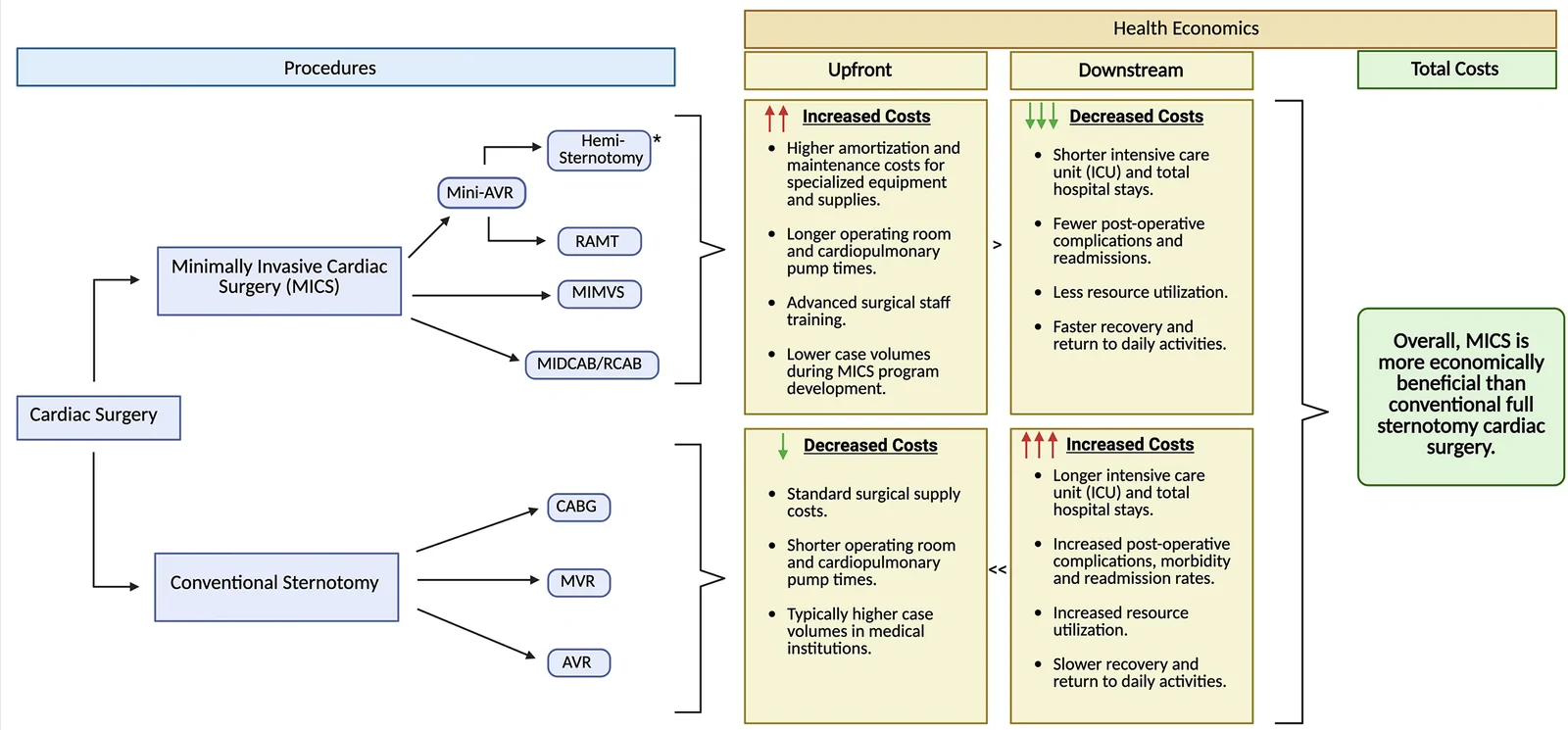
**Schematic comparing the health economics of MICS with conventional sternotomy approaches**. Although multiple factors contribute to higher upfront costs in most MICS procedures, downstream economic advantages often translate into lower overall procedural costs. * indicates that hemi-sternotomy AVR is an exception to this trend, as current evidence does not demonstrate consistent cost‐saving benefits. up arrows, increased costs; down arrows, decreased costs. Graphics were generated using BioRender.

## 6. Current Gaps

A major limitation in the current literature is the absence of comprehensive economic modeling frameworks that capture the full lifecycle costs of MICS technologies. Likely the biggest limitation is the paucity of available publications, particularly those that quantify both procedural and postoperative economic expenditures. Additional constraints include the limited availability of Canadian-specific data, which hinders the generalizability of international findings to publicly funded healthcare systems.

Key gaps in the field also include minimal reporting on total cost expenditure for MICS procedures and equipment, insufficient longitudinal data, and heterogeneity in economic reporting, all of which limit meaningful cross-study comparisons. Moreover, the health-economic performance of MICS is highly context-dependent, influenced by the structure of the healthcare system, whether public, private, or hybrid, and by institutional factors such as case volume, institutional perioperative workflow, and surgeon experience. Therefore, cost-effectiveness observed in one country or center may not directly translate to another setting without careful adaptation to local policies, reimbursement models, and resource availability.

Differences in reported cost-effectiveness are attributable to not only procedural volume and experience levels at different institutes, but also to impactful variation in cost-accounting methodology. This review of literature has demonstrated that many studies evaluate only direct hospital expenditures while excluding fixed costs such as robotic platform depreciation, maintenance contracts and infrastructure investment. The consequence of this is that studies may overestimate the economic advantage of robotic surgery. This emphasizes the need for future health economic studies to standardize costing methodologies to obtain an even more accurate reflection of the true value of adopting MICS.

It is also important to recognize that the evidence base surrounding MICS remains relatively limited. Although numerous studies suggest that MICS can offer clinical advantages, these are often balanced out by steep initial investment costs. As such, randomized trials have not been able to consistently demonstrate superiority of MICS over conventional procedures. Furthermore, a large portion of current literature is derived from cohort studies, which are inherently susceptible to selection and performance biases. In the context of this review, efforts to at least demonstrate the non-inferiority of MICS compared to conventional procedures may influence perioperative management, such as shortening hospital and ICU lengths of stay, thereby shifting the results to favour MICS. Taking this into consideration, current literature should be approached with caution as methodological differences and potential sources of bias may significantly influence reported outcomes.

Future studies on the healthcare economics of MICS should incorporate cost-utility analyses (e.g., QALYs), standardized longitudinal cost-benefit models, and societal-perspective analyses, while explicitly contextualizing findings within the framework of the relevant healthcare system. In addition to reporting metrics like QALYs, future studies would also benefit from reporting incremental cost-effective ratio (ICER) analyses using country-specific thresholds to determine whether MICS can bring good value to country-specific healthcare systems. Where applicable, internationally recognized benchmarks like the World Health Organization (WHO) recommendation of 1–3 times gross domestic product (GDP) per capita or national thresholds may act as useful reference points for interpreting cost-utility outcomes across different healthcare settings. This approach is essential to generate actionable, generalizable evidence for decision-makers considering the adoption or expansion of MICS programs. Future economic evaluations should also adopt Time-Driven Activity-Based Costing (TDABC) methodologies to more accurately characterize resource utilization across the whole perioperative pathway. TDABC has the potential to identify major cost drivers, improve transparency within resource allocation, and facilitate meaningful comparisons between practices across multiple different healthcare institutions.

Finally, future economic analyses should consider the evolving role of transcatheter structural heart interventions such as Transcatheter Aortic Valve Replacement (TAVR) and MitraClip. As the indications for these less invasive, non-surgical interventions continue to expand, the need for comparisons between MICS and transcatheter procedures increases to determine the superior alternative in terms of long-term clinical outcomes, quality of life, durability, reintervention rates, and overall cost-effectiveness.

## 7. Conclusion

Overall, MICS appears to be a strategic, forward-looking investment for healthcare systems aiming to enhance efficiency, improve patient outcomes, and optimize resource utilization. Intraoperative costs remain substantial, driven largely by evolving robotic technologies and specialized instrumentation. However, these expenses are frequently offset by meaningful postoperative and societal savings. When implemented in high-volume, well-standardized centers, MICS demonstrates strong potential for long-term cost-effectiveness and system-level gains. As health systems place greater emphasis on value-based care, the adoption of MICS within cardiac surgery centers and training programs must be strategic, ensuring that investments are justified by patient outcomes and overall healthcare economic sustainability.

## Abbreviations

AVR, Aortic Valve Replacement; CABG, Coronary Artery Bypass Grafting; ERAS, Enhanced Recovery After Surgery; ICU, Intensive Care Unit; LAD, Left Anterior Descending; MeSH, Medical Subject Headings; MICS, Minimally Invasive Cardiac Surgery; MIDCAB, Minimally Invasive Direct Coronary Artery Bypass; MIMVS, Minimally Invasive Mitral Valve Surgery; Mini-AVR, Minimally Invasive Aortic Valve Replacement; NHS, National Health Service; QALYs, Quality-Adjusted Life Years; RAMT, Right Anterior Mini Thoracotomy; RCAB, Robotic-Assisted Coronary Artery Bypass.

## References

[1] Sittimart M, Rattanavipapong W, Mirelman AJ, Hung TM, Dabak S, Downey LE (2024). An overview of the perspectives used in health economic evaluations. Cost Effectiveness and Resource Allocation : C/E.

[2] Wagner TH, Hoomans T, Salloum RG, Levy DE (2025). Efficiency in health care: connecting economic evaluations with implementation objectives. Implementation Science Communications.

[3] Petrou S, Wolstenholme J (2000). A review of alternative approaches to healthcare resource allocation. PharmacoEconomics.

[4] Ilcheva L, Risteski P, Tudorache I, Häussler A, Papadopoulos N, Odavic D (2023). Beyond Conventional Operations: Embracing the Era of Contemporary Minimally Invasive Cardiac Surgery. Journal of Clinical Medicine.

[5] Wilbring M (2025). Advancing Minimally Invasive Cardiac Surgery-Let's Take a Look into the Future. Journal of Clinical Medicine.

[6] Raja SG (2025). New Clinical Advances in Minimally Invasive Coronary Surgery. Journal of Clinical Medicine.

[7] Karangelis D, Androutsopoulou V, Tzifa A, Chalikias G, Tziakas D, Mitropoulos F (2021). Minimally invasive cardiac surgery: in the pursuit to treat more and hurt less. Journal of Thoracic Disease.

[8] Gregory AJ, Kent WDT, Adams C, Arora RC (2024). Closing the care gap: combining enhanced recovery with minimally invasive valve surgery. Current Opinion in Cardiology.

[9] Jung JC, Kim KH (2016). Minimally Invasive Cardiac Surgery versus Conventional Median Sternotomy for Atrial Septal Defect Closure. The Korean Journal of Thoracic and Cardiovascular Surgery.

[10] Modi P, Hassan A, Chitwood WR (2008). Minimally invasive mitral valve surgery: a systematic review and meta-analysis. European Journal of Cardio-thoracic Surgery : Official Journal of the European Association for Cardio-thoracic Surgery.

[11] Piekarski F, Rohner M, Monsefi N, Bakhtiary F, Velten M (2024). Anesthesia for Minimal Invasive Cardiac Surgery: The Bonn Heart Center Protocol. Journal of Clinical Medicine.

[12] İyigün T, Kaya M, Gülbeyaz SÖ, Fıstıkçı N, Uyanık G, Yılmaz B (2017). Patient body image, self-esteem, and cosmetic results of minimally invasive robotic cardiac surgery. International Journal of Surgery (London, England).

[13] Aston D, Zeloof D, Falter F (2023). Anaesthesia for Minimally Invasive Cardiac Surgery. Journal of Cardiovascular Development and Disease.

[14] Ammannaya GKK, Solinas M, Passino C (2020). Analysis of the logistical, economic and minimally invasive cardiac surgical training difficulties in India. Archives of Medical Sciences. Atherosclerotic Diseases.

[15] Sembo TWS, Fatehi Hassanabad A, Stevens K, Brown AN, Parhar K, Adams C (2025). Cost Analysis for Minimally Invasive Mitral Valve Surgery: A Single-Centre Canadian Study. CJC Open.

[16] Coast J (1993). The role of economic evaluation in setting priorities for elective surgery. Health Policy (Amsterdam, Netherlands).

[17] Javidan AP, Naji F, Li A, Wu A, Srivatsav V, Rapanos T (2020). A Systematic Review of Economic Evaluations in Vascular Surgery. Annals of Vascular Surgery.

[18] Eseonu K, Oduoza U, Monem M, Tahir M (2022). Systematic Review of Cost-Effectiveness Analyses Comparing Open and Minimally Invasive Lumbar Spinal Surgery. International Journal of Spine Surgery.

[19] Mayol AJ (2022). Value-based Surgery. Journal of Healthcare Quality Research.

[20] Chang D, Zygourakis CC, Wadhwa H, Kahn JG (2020). Systematic Review of Cost-Effectiveness Analyses in U.S. Spine Surgery. World Neurosurgery.

[21] Seo YJ, Sareh S, Hadaya J, Sanaiha Y, Ziaeian B, Shemin RJ (2022). Factors Associated With High Resource Use in Elective Adult Cardiac Surgery From 2005 to 2016. The Annals of Thoracic Surgery.

[22] Greco G, Shi W, Michler RE, Meltzer DO, Ailawadi G, Hohmann SF (2015). Costs associated with health care-associated infections in cardiac surgery. Journal of the American College of Cardiology.

[23] Brown AN, Stevens K, Kim T, Hemmati S, Koury H, Sandhu J (2025). A Single-Centre Canadian Cost Analysis for the Care and Management of Injection Drug Use-Associated Infective Endocarditis Requiring Cardiac Surgery. CJC Open.

[24] Teman NR, Hawkins RB, Charles EJ, Mehaffey JH, Speir AM, Quader MA (2021). Minimally Invasive vs Open Coronary Surgery: A Multi-Institutional Analysis of Cost and Outcomes. The Annals of Thoracic Surgery.

[25] Ferraris VA, Ferraris SP (2001). Cost-effectiveness of minimally invasive cardiac operations. The Heart Surgery Forum.

[26] Reichenspurner H, Boehm D, Detter C, Schiller W, Reichart B (1999). Economic evaluation of different minimally invasive procedures for the treatment of coronary artery disease. European Journal of Cardio-thoracic Surgery : Official Journal of the European Association for Cardio-thoracic Surgery.

[27] Arom KV, Emery RW, Flavin TF, Petersen RJ (1999). Cost-effectiveness of minimally invasive coronary artery bypass surgery. The Annals of Thoracic Surgery.

[28] Gianoli M, de Jong AR, van der Harst P, van der Kaaij NP, Jacob KA, Suyker WJL (2024). Cost Analysis of Robot-Assisted Versus On-Pump and Off-Pump Coronary Artery Bypass Grafting: A Single-Center Surgical and 30-Day Outcomes Comparison. Innovations (Philadelphia, Pa.).

[29] Lam K, Clarke J, Purkayastha S, Kinross JM (2021). Uptake and accessibility of surgical robotics in England. The International Journal of Medical Robotics + Computer Assisted Surgery : MRCAS.

[30] Barbash GI, Glied SA (2010). New technology and health care costs--the case of robot-assisted surgery. The New England Journal of Medicine.

[31] Hancock HC, Maier RH, Kasim A, Mason J, Murphy G, Goodwin A (2021). Mini-sternotomy versus conventional sternotomy for aortic valve replacement: a randomised controlled trial. BMJ Open.

[32] Nair SK, Sudarshan CD, Thorpe BS, Singh J, Pillay T, Catarino P (2018). Mini-Stern Trial: A randomized trial comparing mini-sternotomy to full median sternotomy for aortic valve replacement. The Journal of Thoracic and Cardiovascular Surgery.

[33] Aasim M, Aziz R, Mohsin AU, Khan R, Aziz G, Ikram J (2024). Comparison of the Outcomes of Aortic Valve Replacements (AVRs) Performed via Conventional Full Sternotomy and Upper Mini Sternotomy: Our Experience at Hayatabad Medical Complex, Pakistan. Cureus.

[34] Kirmani BH, Knowles A, Saravanan P, Zacharias J (2021). Establishing minimally invasive cardiac surgery in a low-volume mitral surgery centre. Annals of the Royal College of Surgeons of England.

[35] Sanad M, Beshir H (2020). Minimally invasive aortic valve replacement with central cannulation: A cost-benefit analysis in a developing country. The Cardiothoracic Surgeon.

[36] Rodriguez E, Malaisrie SC, Mehall JR, Moore M, Salemi A, Ailawadi G (2014). Right anterior thoracotomy aortic valve replacement is associated with less cost than sternotomy-based approaches: a multi-institution analysis of 'real world' data. Journal of Medical Economics.

[37] Ghanta RK, Lapar DJ, Kern JA, Kron IL, Speir AM, Fonner E (2015). Minimally invasive aortic valve replacement provides equivalent outcomes at reduced cost compared with conventional aortic valve replacement: A real-world multi-institutional analysis. The Journal of Thoracic and Cardiovascular Surgery.

[38] Hassan M, Miao Y, Maraey A, Lincoln J, Brown S, Windsor J (2015). Minimally Invasive Aortic Valve Replacement: Cost-Benefit Analysis of Ministernotomy Versus Minithoracotomy Approach. The Journal of Heart Valve Disease.

[39] Santana O, Larrauri-Reyes M, Zamora C, Mihos CG (2016). Is a minimally invasive approach for mitral valve surgery more cost-effective than median sternotomy?. Interactive Cardiovascular and Thoracic Surgery.

[40] McGinn JT, Usman S, Lapierre H, Pothula VR, Mesana TG, Ruel M (2009). Minimally invasive coronary artery bypass grafting: dual-center experience in 450 consecutive patients. Circulation.

[41] Bonatti J, Wallner S, Crailsheim I, Grabenwöger M, Winkler B (2021). Minimally invasive and robotic coronary artery bypass grafting-a 25-year review. Journal of Thoracic Disease.

[42] Wu Y, Ren B, Li J, Chi L, Li P, Wu J (2025). Clinical Characteristics and Cardiac Rehabilitation Outcomes During the Perioperative Period After MIDCAB and OPCAB Surgery: A Comparative Study. Journal of Cardiovascular Development and Disease.

[43] Yang M, Xiao LB, Gao ZS, Zhou JW (2017). Clinical Effect and Prognosis of Off-Pump Minimally Invasive Direct Coronary Artery Bypass. Medical Science Monitor : International Medical Journal of Experimental and Clinical Research.

[44] Patrick WL, Iyengar A, Han JJ, Mays JC, Helmers M, Kelly JJ (2021). The learning curve of robotic coronary arterial bypass surgery: A report from the STS database. Journal of Cardiac Surgery.

[45] Stringfield SB, Parry LA, Eisenstein SG, Horgan SN, Kane CJ, Ramamoorthy SL (2022). Experience with 10 years of a robotic surgery program at an Academic Medical Center. Surgical Endoscopy.

[46] Turchetti G, Palla I, Pierotti F, Cuschieri A (2012). Economic evaluation of da Vinci-assisted robotic surgery: a systematic review. Surgical Endoscopy.

[47] van der Sluis PC, Schreuder HWR, Merks BT, Boeken Kruger AE, Verheijen RHM, van Hillegersberg R (2013). Centralization of robotic surgery: better results and cost savings. Nederlands Tijdschrift Voor Geneeskunde.

[48] Schreyögg J, Stargardt T (2010). The trade-off between costs and outcomes: the case of acute myocardial infarction. Health Services Research.

[49] Salamate S, Bakhtiary F, Bayram A, Sirat S, Doss M, Ciobanu V (2024). Comparison of Two Surgical Approaches for Coronary Artery Bypass of Left Anterior Descending Artery. Journal of Clinical Medicine.

[50] Shen C, Gu D, Klein R, Zhou S, Shih YCT, Tracy T (2020). Factors Associated With Hospital Decisions to Purchase Robotic Surgical Systems. MDM Policy & Practice.

[51] Khalid S, Hassan M, Ali A, Anwar F, Siddiqui MS, Shrestha S (2024). Minimally invasive approaches versus conventional sternotomy for aortic valve replacement in patients with aortic valve disease: a systematic review and meta-analysis of 17 269 patients. Annals of Medicine and Surgery (2012).

[52] Kirmani BH, Jones SG, Muir A, Malaisrie SC, Chung DA, Williams RJ (2023). Limited versus full sternotomy for aortic valve replacement. The Cochrane Database of Systematic Reviews.

[53] Fatehi Hassanabad A, King MA, Karolak W, Dokollari A, Castejon A, de Waard D (2024). Right Anterior Minithoracotomy Approach for Aortic Valve Replacement. Innovations (Philadelphia, Pa.).

[54] Ribeiro IB, Ruel M (2019). Right Anterior Minithoracotomy for Aortic Valve Replacement: A Widely Applicable, Simple, and Stepwise Approach. Innovations (Philadelphia, Pa.).

[55] Dost B, Turunc E, Aydin ME, Kaya C, Aykut A, Demir ZA (2025). Pain Management in Minimally Invasive Cardiac Surgery: A Review of Current Clinical Evidence. Pain and Therapy.

[56] Hawkins RB, Mehaffey JH, Kessel SM, Dahl JJ, Kron IL, Kern JA (2018). Minimally invasive mitral valve surgery is associated with excellent resource utilization, cost, and outcomes. The Journal of Thoracic and Cardiovascular Surgery.

[57] Perin G, Shaw M, Toolan C, Palmer K, Al-Rawi O, Modi P (2021). Cost Analysis of Minimally Invasive Mitral Valve Surgery in the UK National Health Service. The Annals of Thoracic Surgery.

[58] Moss E, Halkos ME (2017). Cost effectiveness of robotic mitral valve surgery. Annals of Cardiothoracic Surgery.

[59] Abbas A, Bakhos C, Petrov R, Kaiser L (2020). Financial impact of adapting robotics to a thoracic practice in an academic institution. Journal of Thoracic Disease.

[60] Calabrò RS, Müller-Eising C, Diliberti ML, Manuli A, Parrinello F, Rao G (2020). Who Will Pay for Robotic Rehabilitation? The Growing Need for a Cost-effectiveness Analysis. Innovations in Clinical Neuroscience.

[61] Landi S, Maistri G, Orsini LP, Leardini C, Malandra S, Antonelli A (2025). Supporting managerial decisions: a comparison of new robotic platforms through time-driven activity-based costing within a value-based healthcare framework. BMC Health Services Research.

[62] Probst P (2023). A Review of the Role of Robotics in Surgery: To DaVinci and Beyond!. Missouri Medicine.

[63] Kofidis T, Chang G (2020). How to set up a minimally invasive cardiac surgery program?. Turk Gogus Kalp Damar Cerrahisi Dergisi.

[64] Ganushchak Y, Nia PS (2018). Perfusion techniques in minimally invasive setting. Journal of Visualized Surgery.

[65] Onan B (2020). Minimal access in cardiac surgery. Turk Gogus Kalp Damar Cerrahisi Dergisi.

[66] Steeg K, Krombach GA, Friebe MH (2025). A Review of Needle Navigation Technologies in Minimally Invasive Cardiovascular Surgeries-Toward a More Effective and Easy-to-Apply Process. Diagnostics (Basel, Switzerland).

[67] Alwatari Y, Randall L, Rowse PG, Daly RC, Arghami A (2024). Navigating missing link: innovative cannulation for robotic mitral valve repair in the absence of inferior vena cava. Journal of Surgical Case Reports.

[68] Kawano Y, Tabata M (2018). Surgical Devices for Minimally Invasive Cardiac Surgery(MICS). Kyobu Geka. the Japanese Journal of Thoracic Surgery.

[69] Faerber G, Mukharyamov M, Doenst T (2023). Is There a Future for Minimal Access and Robots in Cardiac Surgery?. Journal of Cardiovascular Development and Disease.

[70] Iribarne A, Easterwood R, Russo MJ, Wang YC, Yang J, Hong KN (2011). A minimally invasive approach is more cost-effective than a traditional sternotomy approach for mitral valve surgery. The Journal of Thoracic and Cardiovascular Surgery.

[71] Petersen J, Kloth B, Konertz J, Kubitz J, Schulte-Uentrop L, Ketels G (2021). Economic impact of enhanced recovery after surgery protocol in minimally invasive cardiac surgery. BMC Health Services Research.

[72] Ong ZX, Wu D, Luo H, Chang G, Sazzad F, Sule JA (2022). Comparison of the safety and efficacy between minimally invasive cardiac surgery and median sternotomy in a low-risk mixed Asian population in Singapore. Singapore Medical Journal.

[73] Yang NK, Soliman FK, Pepe RJ, Palte NK, Yoo J, Nithikasem S (2023). Minimally invasive approach associated with lower resource utilization after aortic and mitral valve surgery. JTCVS Open.

[74] Zhang X, Wang C, Yu H, Song Y, He Y, Zhao T (2025). A survey on perioperative antibiotic use for minimally invasive coronary artery bypass grafting. General Thoracic and Cardiovascular Surgery.

[75] Poston RS, Tran R, Collins M, Reynolds M, Connerney I, Reicher B (2008). Comparison of economic and patient outcomes with minimally invasive versus traditional off-pump coronary artery bypass grafting techniques. Annals of Surgery.

[76] Downs EA, Johnston LE, LaPar DJ, Ghanta RK, Kron IL, Speir AM (2016). Minimally Invasive Mitral Valve Surgery Provides Excellent Outcomes Without Increased Cost: A Multi-Institutional Analysis. The Annals of Thoracic Surgery.

[77] Grossi EA, Goldman S, Wolfe JA, Mehall J, Smith JM, Ailawadi G (2014). Minithoracotomy for mitral valve repair improves inpatient and postdischarge economic savings. The Journal of Thoracic and Cardiovascular Surgery.

[78] Holzhey DM, Seeburger J, Misfeld M, Borger MA, Mohr FW (2013). Learning minimally invasive mitral valve surgery: a cumulative sum sequential probability analysis of 3895 operations from a single high-volume center. Circulation.

[79] Ali JM, Abu-Omar Y (2021). Minimally invasive cardiac surgery-a Fad or the Future?. Journal of Thoracic Disease.

[80] Ho V, Petersen LA (2007). Estimating cost savings from regionalizing cardiac procedures using hospital discharge data. Cost Effectiveness and Resource Allocation : C/E.

[81] Scarborough OM, Shah S, Scarborough AJ, Deshpande R (2025). Patient Satisfaction With Minimally Invasive Cardiac Incisions: A Retrospective Single-Centre Survey. Cureus.

[82] Whiteley J, Toolan C, Shaw M, Perin G, Palmer K, Al-Rawi O (2021). Patient-reported outcome measures after mitral valve repair: a comparison between minimally invasive and sternotomy. Interactive Cardiovascular and Thoracic Surgery.

[83] Moscarelli M, Lorusso R, Abdullahi Y, Varone E, Marotta M, Solinas M (2021). The Effect of Minimally Invasive Surgery and Sternotomy on Physical Activity and Quality of Life. Heart, Lung & Circulation.

